# Administrative Frictions and the Mental Health Workforce

**DOI:** 10.1001/jamahealthforum.2024.0207

**Published:** 2024-03-01

**Authors:** Jane M. Zhu, Matthew Eisenberg

**Affiliations:** Division of General Internal Medicine, Oregon Health & Science University, Portland; Center for Mental Health and Addiction Policy, Department of Health Policy and Management, Bloomberg School of Public Health, Johns Hopkins University, Baltimore, Maryland

Treatment gaps continue to grow for US individuals with mental health conditions. A third of those with any mental illness and half of those with serious mental illness reported unmet needs in 2021.^1^ Despite improvements in coverage for mental health care over the past 2 decades, demand for services continues to increase and access has lagged. Even insured consumers face significant challenges in finding available and accessible in-network practitioners due to mental health professionals’ low participation rates in public and private insurance networks. Many factors disincentivize network participation, including low reimbursement rates, clinical complexity and acuity, and insufficient workplace supports.^2^ Yet, 1 factor often overlooked is the hefty burden of administrative barriers facing mental health professionals along multiple points of the clinical practice continuum.

Compared with those in medical and surgical specialties, the mental health workforce is less likely to accept insurance and to participate in health plan networks. Empirical evidence has been limited largely to psychiatrists, though the gray literature suggests similar issues for other specialty mental health professionals (psychiatric nurse practitioners, psychologists, therapists, counselors, and licensed clinical social workers). Existing evidence is glaring: nearly 50% of psychiatrists do not accept any form of insurance compared with 20% of physicians across other specialties,^3^ a trend increasing over time. Substantial differences exist across payers; only 35% of psychiatrists accepted new Medicaid enrollees in 2014 and 2015 compared with 62% for patients covered by Medicare and private insurance.^4^ Given that public payers finance half of all mental health spending, these disparities can lead to delayed care and inadequate treatment access. Patients with cost-sharing options to go out of network for care also accrue higher out-of-pocket costs for mental health than for medical and surgical services.^5^

The reasons for low insurance participation rates are complex, historical, and structural. A persistent supply and demand imbalance, combined with a willingness by some patients to pay out-of-pocket to see their preferred practitioners, affords practitioners substantial market power to select where and how they practice. The success of cash pay private practice models,^6^ with the cost for psychotherapy ranging between $100 and $300 per session, magnifies persistent reimbursement disparities within insurance systems. Not only are mental health professionals paid at lower rates than clinicians in other specialties for the same services, but they are paid 20% less for these services by Medicaid compared with Medicare.^7^ In commercial and Medicare Advantage markets, mental health professionals are paid approximately half as much for services delivered in network vs out of network.^5^

Research suggests, however, that administrative burdens play as important a role as reimbursement in influencing clinicians’ decisions to accept insurance.^8^ While practitioners in other specialties face similar administrative burdens, the majority of mental health professionals operate solo and small practices. As a result, they often lack the significant financial, operational, and administrative support needed for claims processing, revenue cycle management, and health information technology. One study estimated that Medicaid-participating physicians lose 18% of Medicaid revenue to billing problems, particularly through repeated claims denials and resubmissions, and that these costs are as important as payment rates for explaining variation in willingness to accept Medicaid.^8^ Such activities directly cut into clinical practice time, contribute to burnout, and are associated with poorer practitioner and organizational performance.^2^ These burdens multiply if practitioners contract with multiple health plans with their own data collection processes.

Other claims-related burdens are pervasive, including coding and billing limitations around types of reimbursable services and practitioner eligibility, which often do not reflect mental health professionals’ scope of services and vary across payers. In some states, complex billing rules exist for clients with co-occurring mental health and substance use disorders, including requirements for practitioners to be dual credentialed and for separate prior authorization processes for substance use and mental health services. Payers can also set medical necessity determinations, treatment plans, and limits on the duration of covered treatments that further complicate claims management.

Additional administrative burdens may be unique to mental health delivery. For instance, new practitioners seeking to participate in a health plan network must undergo a credentialing and contracting process that can take multiple months. These processes sometimes diverge from medical practitioners’ contracting processes, with different administrative oversight, documentation, and timelines across plans. Furthermore, payers that partner with third-party behavioral health organizations to deliver services—a common arrangement in Medicaid managed care—may introduce even more administrative complexities to clinicians who must interface with an entirely distinct organization.

Lastly, administrative and reporting requirements hinder day-to-day care delivery. To receive full reimbursement in Medicaid, for example, mental health professionals often are required to conduct an intake appointment, requiring practitioners to conduct an extended assessment, write a treatment plan, and make a diagnosis before any services can be delivered. Given that follow-up psychopharmacologic evaluation can take weeks longer to schedule, delays may make the difference between crisis stabilization and escalation for those needing immediate treatment.^9^ Moreover, given the fragmentation between physical and mental health systems, nonintegrated technology systems that lack interoperability may make it difficult to track clients across settings or to locate historical documents necessary for multiple assessments and reports to insurers.

Public administration experts have long highlighted the costs that administrative burdens pose.^10^ Learning costs refer to the challenges of learning how to navigate system complexity. Psychological costs are associated with stress and loss of autonomy. Compliance costs mean wasted time, energy, and work as a result of efforts needed to adhere to administrative rules. In the sphere of public health, it is well understood that these costs deter eligible people from participating in social safety net programs like Medicaid. However, these burdens can have similar effects on the behavioral health workforce. The sum of these parts is an understandable, if unfortunate, conclusion: participation in insurance networks currently relies on practitioners’ goodwill, as it comes with significant administrative hurdles at lower reimbursement than the alternative of cash pay. As the share of mental health professionals accepting insurance continues to decline, policies are critically needed that incentivize and sustain participation in health insurance programs.

What would this take? Beyond addressing reimbursement disparities, multiple levels of state and federal partnerships are needed to promote thorough clinical assessment while minimizing unnecessary administrative repetitions and delays. Standardization of contracting requirements and simplification of claims processes could be initiated at the federal level through Centers for Medicare & Medicaid Services guidelines, which could be adopted and implemented by state-level regulators. The Centers for Medicare & Medicaid Services could work to expand guidance to states on billing eligibility across a range of practitioners and services as well as on payment delays and denial. Insurers could adopt and streamline process automation to increase efficiencies for practitioners by improving options for submitting required information and simplifying paperwork burden. Optum Behavioral Health, for example, implemented an Express Access program, which allows mental health professionals to be added to the network within 7 to 10 days in exchange for initial appointment openings within 5 days of joining. A single, standardized, 2-page form is completed, signed, and submitted in real-time online. Finally, initiatives to expedite credentialing could be supported by state legislation that sets maximum time frames for health plan credentialing processes.

Recent policy proposals have sought to address mental health practitioner shortages in the long term. But, without specific attention to the factors affecting network participation in the shorter term, workforce burnout and attrition will likely persist. Administrative frictions are costly for mental health practitioners and exacerbate ongoing service gaps; addressing these issues may help to incentivize greater workforce participation and retention in insurance markets and make the practitioner base more accessible to a broader swath of individuals in the US.
